# The Kinetics of Humoral and Cellular Responses after the Booster Dose of COVID-19 Vaccine in Inflammatory Arthritis Patients

**DOI:** 10.3390/v15030620

**Published:** 2023-02-24

**Authors:** Jakub Wroński, Bożena Jaszczyk, Leszek Roszkowski, Anna Felis-Giemza, Krzysztof Bonek, Anna Kornatka, Magdalena Plebańczyk, Tomasz Burakowski, Barbara Lisowska, Brygida Kwiatkowska, Włodzimierz Maśliński, Małgorzata Wisłowska, Magdalena Massalska, Ewa Kuca-Warnawin, Marzena Ciechomska

**Affiliations:** 1 Department of Rheumatology, National Institute of Geriatrics, Rheumatology and Rehabilitation, 02-637 Warsaw, Poland; 2 Department of Outpatient Clinics, National Institute of Geriatrics, Rheumatology and Rehabilitation, 02-637 Warsaw, Poland; 3 Biologic Therapy Center, National Institute of Geriatrics, Rheumatology and Rehabilitation, 02-637 Warsaw, Poland; 4 Department of Pathophysiology and Immunology, National Institute of Geriatrics, Rheumatology and Rehabilitation, 02-637 Warsaw, Poland; 5 Department of Anesthesiology, National Institute of Geriatrics, Rheumatology and Rehabilitation, 02-637 Warsaw, Poland; 6 Department of Early Arthritis, National Institute of Geriatrics, Rheumatology and Rehabilitation, 02-637 Warsaw, Poland

**Keywords:** COVID-19, booster vaccine, kinetics, humoral response, cellular response, arthritis

## Abstract

Impaired immunogenicity of COVID-19 vaccinations in inflammatory arthritis (IA) patients results in diminished immunity. However, optimal booster vaccination regimens are still unknown. Therefore, this study aimed to assess the kinetics of humoral and cellular responses in IA patients after the COVID-19 booster. In 29 IA patients and 16 healthy controls (HC), humoral responses (level of IgG antibodies) and cellular responses (IFN-γ production) were assessed before (T0), after 4 weeks (T1), and after more than 6 months (T2) from the booster vaccination with BNT162b2. IA patients, but not HC, showed lower anti-S-IgG concentration and IGRA fold change at T2 compared to T1 (*p* = 0.026 and *p* = 0.031). Furthermore, in IA patients the level of cellular response at T2 returned to the pre-booster level (T0). All immunomodulatory drugs, except IL-6 and IL-17 inhibitors for the humoral and IL-17 inhibitors for the cellular response, impaired the immunogenicity of the booster dose at T2. Our study showed impaired kinetics of both humoral and cellular responses after the booster dose of the COVID-19 vaccine in IA patients, which, in the case of cellular response, did not allow the vaccination effect to be maintained for more than 6 months. Repetitive vaccination with subsequent booster doses seems to be necessary for IA patients.

## 1. Introduction

Inflammatory arthritis (IA) patients treated with immunomodulatory drugs are among the vulnerable immune-incompetent patients at increased risk of COVID-19 [1,2,3] and increased COVID-19 mortality [3]. For this reason, vaccinations play a crucial role in the prevention of COVID-19 among patients with IA. However, previous studies have established impaired immunogenicity of COVID-19 vaccinations in IA patients after the primary vaccination schedule. Studies, focusing mostly on the post-vaccination humoral response, proved both lower antibody levels in IA patients in comparison to healthy controls (HC) [4,5,6,7,8] and an increased risk of a complete lack of humoral response [4,9,10,11,12,13,14,15,16]. Subsequent studies have also proven a reduction in the cellular response to primary COVID-19 vaccinations in patients with IA [17,18]. Immunomodulatory drugs used by IA patients are seen as the main reason for the reduced post-vaccination immune response. Immunomodulatory drugs that reduce the immunogenicity of COVID-19 vaccines to the greatest extent are rituximab [4,6,8,9,10,13,14,17,19,20,21,22], glucocorticosteroids (GCs) [4,10,12,14,15,16,19,21], and abatacept [13,14,16,22,23], with single studies, also showing a decrease in the humoral response after methotrexate (MTX) [8,11,12,14,15], TNF inhibitors [7,15,22,24], IL-6 inhibitors [23], and JAK inhibitors [8,22].

The abrogated immunological response to the primary vaccination schedule results in a faster decline of post-vaccination immunity in IA patients [5,7,16,18,25,26], making booster vaccinations among IA patients even more important than in HC. Current studies proved that, though COVID-19 booster vaccinations are effective in IA patients [27,28,29], also the immunogenicity of the booster dose is impaired in this group of patients [15,30,31,32,33,34,35,36]. There are only single studies evaluating the effect of immunomodulatory drugs on the immunogenicity of the booster dose against COVID-19, indicating worsen response after GCs [30], biological and targeted synthetic disease-modifying antirheumatic drugs (bDMARDs and tsDMARDs) [15,30], and MTX [34,35]. In our previous study, we assessed the effect of immunomodulatory drugs on humoral and cellular responses after the booster dose [36]. The humoral response after the booster dose was likewise in other studies only impaired by bDMARDs and tsDMARDs, but the cellular response was decreased after all immunomodulatory drugs except IL-17 inhibitors and sulfasalazine.

Although studies show reduced immunogenicity of COVID-19 booster vaccinations among IA patients, which suggests the need for more frequent booster vaccinations among IA patients than among HC, the optimal booster vaccination regimens are still unknown. This is due to the lack of data regarding the kinetics of immunological response after the COVID-19 booster dose. Therefore, we continued the observation of the previously described cohort [36], aiming to assess the kinetics of humoral and cellular responses in IA patients after the booster dose of the COVID-19 vaccine.

## 2. Materials and Methods

### 2.1. Patients

The study was conducted at the COVID-19 vaccination unit in a rheumatology center. IA patients and HC (sex and age-matched) visiting the vaccination unit between November 2021 to January 2022 were enrolled. The inclusion criteria for both groups included age above 18, willingness to become vaccinated with a booster dose of the COVID-19 vaccination (BNT162b2, Pfizer-BioNTech), and a period longer than 6 months from the end of primary COVID-19 vaccination (except for one patient who received single Johnson & Johnson vaccine as the primary vaccination, the booster vaccination was the third dose of vaccination). The additional inclusion criterion for the IA group was a diagnosis of rheumatoid arthritis (RA) according to the ACR-EULAR 2010 criteria, ankylosing spondylitis (AS) according to modified New York criteria, psoriatic arthritis (PsA) according to CASPAR criteria, or non-radiographic spondyloarthritis (nrSpA) according to ASAS 2010 criteria. The exclusion criteria for both groups were a previous allergic reaction to vaccination against COVID-19, serious adverse events after previous vaccination against COVID-19, or other conditions which, in the opinion of the qualifying physician, constitute a contraindication to vaccination. The additional exclusion criteria in the control group were treated with any kind of immunomodulatory therapy or any medical condition that may, in the opinion of the qualifying physician, lower the patient’s immunity. The study protocol was approved by the hospital bioethics committee (KBT-3/2/2021). All participants signed informed consent for inclusion in the study. The study was conducted according to the Declaration of Helsinki.

### 2.2. Methods

Patient characteristics (including the use of immunomodulatory drugs during the primary vaccination schedule and before the booster vaccination) were collected by qualifying physicians using a structured interview. Data regarding primary COVID-19 vaccinations and COVID-19 infections (both before and after booster vaccination) were gathered from both interviews and the national COVID-19 registry. Additionally, to detect asymptomatic COVID-19 infections, antibodies against SARS-CoV-2 nucleocapsid (N) were measured with SARS-CoV N ELISA Kit (TestLine Clinical Diagnostics, Brno, Czech Republic. Data regarding patient characteristics were blinded to the laboratory staff.

Blood samples were collected before the booster COVID-19 vaccination (T0), 4 weeks after the booster vaccination (T1), and after more than 6 months from the booster dose (T2). Blood samples were collected from all recruited patients (49 IA patients and 47 HC) at T0 and T1, and from part of the participants at T2 (29 IA patients and 16 HC). In the kinetics study, we only included participants with data from all time points.

The humoral immunity was assessed with the level of IgG antibodies against the SARS-CoV-2 Spike (S1) antigen measured with anti-SARS-CoV-2 QuantiVac ELISA (Euroimmun, Lübeck, Germany). The cellular immunity was assessed with the IFN-γ production tested with interferon-gamma release assay (IGRA) Quan-T-Cell SARS-CoV-2 (Euroimmun, Lübeck, Germany). In the first stage, freshly drawn heparinized whole blood was incubated with the S1 antigen of the SARS-CoV-2 virus coated on the bottom of the test tube. Whole blood was also incubated in a second negative control tube (assessment of non-specific background response) and a third positive control tube (assessment of overall T cell response after stimulation). After incubation time (22–24 h), serum plasma was obtained. In the second stage, an ELISA test was performed to measure the secreted IFN-γ in the first step of the test.

### 2.3. Statistics

The compliance of the data with the normal distribution was assessed using the Shapiro–Wilk test. The significance of the observed differences between the two groups was assessed using the Student’s T test for variables with a normal distribution, the Mann–Whitney U test for variables without a normal distribution, and categorical variables the Fisher’s exact test. To assess the kinetics the Friedman test was used, with post hoc analysis with Dunn’s test. The significance of the results after adjusting for confounding factors (listed in Table 1) was checked by linear regression. Statistical analysis was performed using Statistica 13.3 software (StatSoft Polska, Cracow, Poland) and figures were created using GraphPad Prism 6 software (GraphPad Software, San Diego, CA, USA).

## 3. Results

### 3.1. Patients

Patient characteristics are presented in Table 1. There were no significant differences between the groups, apart from the older age of the IA group (*p* = 0.002). In both groups, a similar percentage of subjects had COVID-19 after the booster dose, based on the presence of antibodies against SARS-CoV-2 nucleocapsid.

### 3.2. The Kinetics of Humoral Response

**Figure 1:**
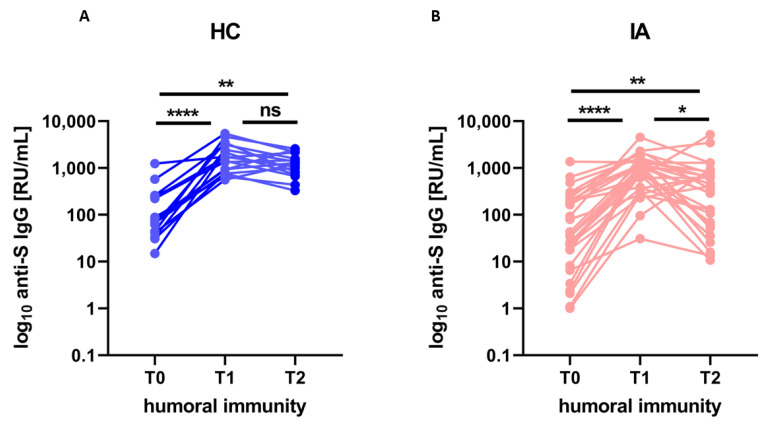
The level of anti-S IgG (humoral immunity) before (T0), after 4 weeks (T1), and after more than 6 months (T2) from the booster dose of the COVID-19 vaccine in (**A**) healthy controls (HC; *n* = 16) compared to (**B**) patients with inflammatory arthritis (IA; *n* = 29). In the group comparison, the Friedman test with Dunn’s multiple comparison test was used. Dots represent individual values and *p* values were expressed as follows: 0.05 > *p* > 0.01 as *, 0.01 > *p* > 0.001 as ** *p* < 0.0001 as ****, ns—nonsignificant.

The kinetics of humoral response (anti-S-IgG concentration) after the COVID-19 booster vaccination is presented in Figure 1. At T2 levels of antibodies were significantly higher in both IA patients and HC than at T0 (*p* = 0.001 and *p* = 0.002 respectively), but lower in IA patients (median 406, min 10.7, max 5166.4) than in HC (median 1253.2, min. 331.4, max. 2561.9). The differences remained significant after adjustment for age (*p* = 0.029). Additionally, in IA, but not in HC, a statistically significant decline (*p* = 0.026) in the humoral response between T1 and T2 was observed, indicating a faster waning of humoral response in IA patients compared to HC.

### 3.3. The Kinetics of Cellular Response

**Figure 2:**
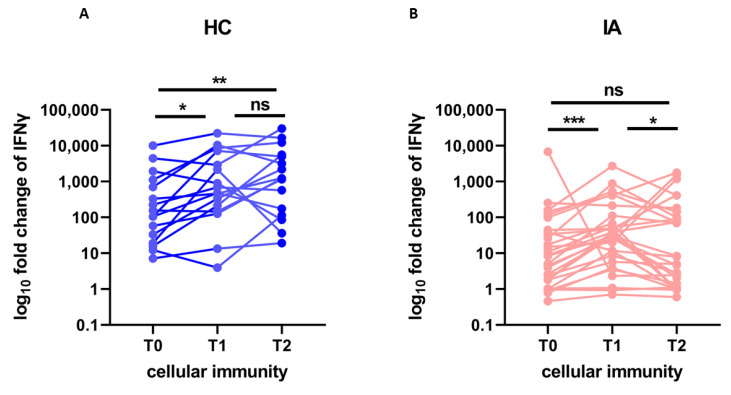
The fold change of IFNγ production stimulated by viral protein (cellular immunity) before (T0), after 4 weeks (T1), and after more than 6 months (T2) from the booster dose of the COVID-19 vaccine in (**A**) healthy controls (HC; *n* = 16) compared to (**B**) patients with inflammatory arthritis (IA; *n* = 29). In the group comparison, the Friedman test with Dunn’s multiple comparison test was used. Dots represent individual values and *p* values were expressed as follows: 0.05 > *p* > 0.01 as *, 0.01 > *p* > 0.001 as **, 0.001 > *p* > 0.0001 as ***, ns—nonsignificant.

The kinetics of cellular response (IGRA fold change) after the COVID-19 booster vaccination is presented in Figure 2. The cellular response at T2 was significantly higher than at T0 in HC (*p* = 0.002), but not significantly different than at T1. However, in IA patients the level of cellular response at T2 was not only significantly lower in IA patients (median fold change 5, min. 0, max. 1763.8) than in HC (median 1236.7, min. 19.2, max. 30,161, after adjustment for age *p* = 0.004). It also significantly decreased from T1 to T2 (*p* = 0.031), returning to the pre-booster level—there was no significant difference between the cellular response at T0 and T2 in IA patients. This fact indicates severely diminished kinetics of the cellular response in IA patients.

### 3.4. The Effect of Immunomodulatory Drugs

The effect of each immunomodulatory drug on the immunogenicity of the COVID-19 vaccines at T2 is shown in Table 2. The analysis showed a significantly reduced humoral response in IA patients compared to HC after all drugs except IL-6 and IL-17 inhibitors and cellular response after all drugs except IL-17 inhibitors. All results of immunomodulatory drugs’ effect after the adjustment for age remained significant. There were no statistically significant differences in humoral and cellular responses between patients using bDMARDs and tsDMARDs in monotherapy vs. in combination with conventional DMARDs. However, a significantly lower cellular (but not humoral) response was observed for IA patients using immunomodulatory drugs in combination with glucocorticoids (*p* < 0.001). The duration of each immunomodulatory treatment was not significantly correlated with the levels of humoral and cellular responses. Interestingly, none of the immunomodulatory drugs affected the kinetics of both humoral and cellular responses after the booster dose (measured as the difference between response rates at T1 and T2).

### 3.5. New COVID-19 Infections

In our relatively small group of patients, we did not observe an effect of the level of humoral and cellular responses at T1 on the chances of new COVID-19 infection in the first 6 months after the booster vaccination, nor did we observe an effect of previous COVID-19 infection on the level of immune response after the booster vaccination.

## 4. Discussion

Our study demonstrates impaired kinetics of both humoral and cellular responses after the booster dose of the COVID-19 vaccine in IA patients. Similarly to our study, a faster decline in the humoral response after a COVID-19 booster dose in patients with IA compared to HC was demonstrated in the study by Mrak et al., although the authors of the study presented only a 12-week follow-up of patients after the booster [37]. In the study by Bjørlykke et al., the authors showed that humoral response to the fourth vaccination in IA patients is even lower than after the third vaccination in HC [38]. The faster decline of the immune response compared to HC after the booster dose indicates the need for more frequent booster vaccinations in patients with IA than in HC. This is in line with the ACR recommendations, which, based on Centers for Disease Control and Prevention recommendations for non-immunocompetent people, currently recommends vaccinating patients with rheumatic diseases with 5 doses of the COVID-19 vaccine in total [39].

The waning of humoral immunity after the COVID-19 booster seems to be slower than after the primary vaccination regimen—the levels of responses were higher at T2 than at T0 and none of the IA patients in our study lost the humoral response after more than 6 months from booster vaccination (compared to 20.4% of IA patients after more than 6 months from the primary schedule as reported in our previous study [36]). We also did not observe any difference in the level of humoral response 6 months after booster vaccination between patients who lost humoral immunity 6 months after primary vaccination and those who maintained it. Furthermore, although most of the immunomodulatory drugs used significantly reduced humoral and cellular response in IA patients compared to HC, both after primary and booster vaccinations, our study did not show their effect on the kinetics of the response. It may indicate that the kinetics impairment may result to a greater extent from dysfunction of the immune system in IA than from immunomodulatory drugs.

Our study, in contrast, to the study by Mrak et al. [37], showed severe impairment in the kinetics of cell-mediated immunity after COVID-19 vaccination. This difference may be due to the shorter period of observation in the aforementioned study. After COVID-19 infection virus-specific T cell half-life is estimated to be of around 200 days [40]. In our IA patients, the level of cellular response after 200 days from the booster dose returned to the pre-booster level (Figure 2). This indicates a severely impaired cellular response in patients with IA and should be taken into account when determining the optimal booster schedules.

The greatest advantage of our study is the assessment of the kinetics of both humoral and cellular responses in IA patients after the booster dose and the effect of individual immunomodulatory drugs on the kinetics. The obvious limitation of our study is a relatively small sample size and unmatched age of the groups (though we accounted for it in our analysis). In particular, the reported effect of individual drugs on the immunogenicity of booster vaccination should be treated with caution. Studies on larger groups of patients may allow the determination of the kinetics with a distinction between various types of IA and to detect (if existent but more subtle) the effect of waning immunity on the rate of COVID-19 infections. Finally, the exact levels of antibodies and cellular responses to protect against COVID-19 are still unknown. In our work, we did not measure the neutralization capacity of antibodies. We measured only anti-S antibody concentration and not RBD which is better correlated with neutralization. We assessed also the cellular response only with IGRA, while interferon-gamma is only one of the components (albeit crucial) of the cellular response.

Our study showed impaired kinetics of immune responses after the booster dose of the COVID-19 vaccine, which, in the case of cellular response, did not allow the vaccination effect to be maintained for more than 6 months. Periodic, more frequent than in HC, vaccinations with subsequent booster doses seem to be necessary for IA patients. Further studies with longer follow-up periods should allow the determination of optimal intervals between vaccinations.

## Figures and Tables

**Table 1:** Patient characteristics. bDMARDs—biological disease-modifying antirheumatic drugs, GCs—glucocorticoids, IL-6i—IL-6 inhibitors, IL-17i—IL-17 inhibitors, JAKi—JAK inhibitors, MTX—methotrexate, N/A—not applicable, nrSpA—non-radiographic spondylarthritis, ns—nonsignificant, SSA—sulfasalazine, TNFi—TNF inhibitors, tsDMARDs—targeted synthetic disease-modifying antirheumatic drugs.

	Inflammatory Arthritis (*n* = 29)	Healthy Control (*n* = 16)	Difference
Age (mean ± SD)	54.5 ± 14.2	41.8 ± 9	*p* = 0.002
Sex—female (*n*, %)	16 (55.2%)	10 (62.5%)	ns
BMI (mean ± SD)	27.2 ± 4.3	27.7 ± 5.6	ns
Smoking (*n*, %) - current - past	3 (10.3%) 8 (27.6%)	1 (6.3%) 1 (6.3%)	ns ns
Vaccine primary scheme (*n*, %) - Pfizer - Moderna - AstraZeneca - Johnson & Johnson	23 (79.3%) 3 (10.3%) 2 (6.9%) 1 (3.5%)	13 (81.2%) 1 (6.3%) 2 (12.5%) -	ns ns ns ns
Heterologous Booster Vaccine (*n*, %)	6 (20.7%)	3 (18.8%)	ns
Days after booster vaccination (median, min, max) - first point (T1) - second point (T2)	31 (22, 52) 201 (167, 290)	31 (27, 77) 199 (175, 234)	ns ns
COVID-19 infection before booster vaccination (*n*, %)	5 (17.2%)	2 (12.5%)	ns
COVID-19 infection after booster vaccination (*n*, %)	5 (17.2%)	3 (18.8%)	ns
**Inflammatory Arthritis (*n* = 29)**			
Disease type (*n*, %) - Rheumatoid arthritis - Ankylosing spondylitis - Psoriatic arthritis - nrSpA	16 (55.2%) 6 (20.7%) 6 (20.7%) 1 (3.4%) 28 (96.6%) 11 (37.9%) 5 (2.5, 37.5) 18 (62.1%) 17 (58.6%) 4 (13.8%) 18 (62.1%) 9 (31%) 5 (17.2%) 2 (6.9%) 2 (6.9%) 11 (37.9%) 7 (24.1%)		
Immunomodulatory treatment during booster (*n*, %)			
GCs (*n*, %) - dose (median, min, max)			
cDMARDs (*n*, %) - MTX - SSA			
bDMARDs and tsDMARDs (*n*, %) - TNFi - JAKi - IL-6i2 - IL-17i bDMARDs and tsDMARDs used: - in monotherapy - with cDMARDs			
Treatment suspension during booster (*n*, %)	2 (6.9%)		

**Table 2:** Effect of immunomodulating drugs on the immunogenicity of the COVID-19 vaccines more than 6 months from booster vaccination. bDMARDs—biological disease-modifying antirheumatic drugs, GCs–glucocorticoids, IL-6i—IL-6 inhibitors, IL-17i—IL-17 inhibitors, JAKi—JAK inhibitors, MTX—methotrexate, ns—nonsignificant, SSZ—sulfasalazine, TNFi—TNF inhibitors, tsDMARDs—targeted synthetic disease-modifying antirheumatic drugs.

IgG Antibodies, Median RU/mL (Min, Max)		
Healthy Control (HC, *n* = 16)	1253.2 (331.4, 2561.9)	The Difference Compared to HC
GCs (*n* = 11)	107.4 (10.7, 1273.4)	*p* < 0.001
MTX (*n* = 17)	406 (10.7, 5166.4)	*p* = 0.007
SSZ (*n* = 4)	452.8 (13.4, 778.1)	*p* = 0.011
bDMARDs and tsDMARDs (*n* = 18)	380.4 (10.7, 3476.4)	*p* < 0.001
- TNFi (*n* = 9)	293 (36.1, 1294.8)	*p* < 0.001
- JAKi (*n* = 5)	614.5 (107.4, 943.2)	*p* = 0.008
- IL-6i (*n* = 2)	351.9 (10.7, 693.1)	ns
- IL-17i (*n* = 2)	1915.6 (354.8, 3476.4)	ns
**IGRA, Median Fold Change (Min, Max)**		
**Healthy Control (HC, *n* = 16)**	**1236.7 (19.2, 30,161)**	**The Difference Compared to HC**
GCs (*n* = 9)	0.95 (0, 182.7)	*p* < 0.001
MTX (*n* = 16)	73.6 (0, 1763.8)	*p* = 0.002
SSZ (*n* = 3)	46.9 (0.9, 142.5)	*p* = 0.016
bDMARDs and tsDMARDs (*n* = 18)	6.3 (0, 1763.8)	*p* < 0.001
- TNFi (*n* = 9)	69.5 (0, 409.7)	*p* < 0.001
- JAKi (*n* = 5)	2.5 (0, 1186.7)	*p* = 0.004
- IL-6i (*n* = 2)	0.85 (0.6, 1.1)	*p* = 0.013
- IL-17i (*n* = 2)	1092.6 (421.4., 1763.8)	ns

## Data Availability

Available upon reasonable request sent to the corresponding author.

## References

[1] Akiyama S., Hamdeh S., Micic D., Sakuraba A. (2020). Prevalence and clinical outcomes of COVID-19 in patients with autoimmune diseases: A systematic review and meta-analysis. Ann. Rheum. Dis..

[2] Wang Q., Liu J., Shao R., Han X., Su C., Lu W. (2021). Risk and clinical outcomes of COVID-19 in patients with rheumatic diseases compared with the general population: A systematic review and meta-analysis. Rheumatol. Int..

[3] Conway R., Grimshaw A.A., Konig M.F., Putman M., Duarte-García A., Tseng L.Y., Cabrera D.M., Chock Y.P., Degirmenci H.B., Duff E. (2022). SARS-CoV-2 Infection and COVID-19 Outcomes in Rheumatic Diseases: A Systematic Literature Review and Meta-Analysis. Arthritis Rheumatol..

[4] Deepak M.P., Kim W., Paley M.A., Yang M., Carvidi B.A.B., Demissie B.E.G., El-Qunni B.A.A., Haile B.A., Huang B.K., Kinnett B.B. (2021). Effect of Immunosuppression on the Immunogenicity of mRNA Vaccines to SARS-CoV-2. Ann. Intern. Med..

[5] Geisen U.M., Sümbül M., Tran F., Berner D.K., Reid H.M., Vullriede L., Ciripoi M., Longardt A.C., Hoff P., Morrison P.J. (2021). Humoral protection to SARS-CoV2 declines faster in patients on TNF alpha blocking therapies. RMD Open.

[6] Seyahi E., Bakhdiyarli G., Oztas M., Kuskucu M.A., Tok Y., Sut N., Ozcifci G., Ozcaglayan A., Balkan I.I., Saltoglu N. (2021). Antibody response to inactivated COVID-19 vaccine (CoronaVac) in immune-mediated diseases: A controlled study among hospital workers and elderly. Rheumatol. Int..

[7] Christensen I.E., Jyssum I., Tveter A.T., Sexton J., Tran T.T., Mjaaland S., Kro G.B., Kvien T.K., Warren D.J., Jahnsen J. (2022). The persistence of anti-Spike antibodies following two SARS-CoV-2 vaccine doses in patients on immunosuppressive therapy compared to healthy controls-a prospective cohort study. BMC Med..

[8] Frommert L.M., de Silva A.N.A., Zernicke J., Scholz V., Braun T., Jeworowski L.M., Schwarz T., Tober-Lau P., Hagen A.T., Habermann E. (2022). Type of vaccine and immunosuppressive therapy but not diagnosis critically influence antibody response after COVID-19 vaccination in patients with rheumatic disease. RMD Open.

[9] Spiera R., Jinich S., Jannat-Khah D. (2021). Rituximab, but not other antirheumatic therapies, is associated with impaired serological response to SARS- CoV-2 vaccination in patients with rheumatic diseases. Ann. Rheum. Dis..

[10] A Ruddy J., Connolly C.M., Boyarsky B.J., A Werbel W., Christopher-Stine L., Garonzik-Wang J., Segev D.L., Paik J.J. (2021). High antibody response to two-dose SARS-CoV-2 messenger RNA vaccination in patients with rheumatic and musculoskeletal diseases. Ann. Rheum. Dis..

[11] Haberman R.H., Herati R., Simon D., Samanovic M., Blank R.B., Tuen M., Koralov S.B., Atreya R., Tascilar K., Allen J.R. (2021). Methotrexate hampers immunogenicity to BNT162b2 mRNA COVID-19 vaccine in immune-mediated inflammatory disease. Ann. Rheum. Dis..

[12] Bugatti S., De Stefano L., Balduzzi S., Greco M.I., Luvaro T., Cassaniti I., Bogliolo L., Mazzucchelli I., D’Onofrio B., di Lernia M. (2021). Methotrexate and glucocorticoids, but not anticytokine therapy, impair the immunogenicity of a single dose of the BNT162b2 mRNA COVID-19 vaccine in patients with chronic inflammatory arthritis. Ann. Rheum. Dis..

[13] Braun-Moscovici Y., Kaplan M., Braun M., Markovits D., Giryes S., Toledano K., Tavor Y., Dolnikov K., Balbir-Gurman A. (2021). Disease activity and humoral response in patients with inflammatory rheumatic diseases after two doses of the Pfizer mRNA vaccine against SARS-CoV-2. Ann. Rheum. Dis..

[14] Furer V., Eviatar T., Zisman D., Peleg H., Paran D., Levartovsky D., Zisapel M., Elalouf O., Kaufman I., Meidan R. (2021). Immunogenicity and safety of the BNT162b2 mRNA COVID-19 vaccine in adult patients with autoimmune inflammatory rheumatic diseases and in the general population: A multicentre study. Ann. Rheum. Dis..

[15] Saad C.G., Silva M.S., Sampaio-Barros P.D., Moraes J.C., Schainberg C.G., Gonçalves C.R., Shimabuco A.Y., Aikawa N.E., Yuki E.F., Pasoto S.G. (2022). Interaction of TNFi and conventional synthetic DMARD in SARS-CoV-2 vaccine response in axial spondyloarthritis and psoriatic arthritis. Joint Bone Spine.

[16] Yamaguchi Y., Nameki S., Kato Y., Saita R., Sato T., Nagao S., Murakami T., Yoshimine Y., Amiya S., Morita T. (2022). Persistence of SARS-CoV-2 neutralizing antibodies and anti-Omicron IgG induced by BNT162b2 mRNA vaccine in patients with autoimmune inflammatory rheumatic disease: An explanatory study in Japan. Lancet Reg. Health West Pac..

[17] Sidler D., Born A., Schietzel S., Horn M.P., Aeberli D., Amsler J., Möller B., Njue L.M., Medri C., Angelillo-Scherrer A. (2022). Trajectories of humoral and cellular immunity and responses to a third dose of mRNA vaccines against SARS-CoV-2 in patients with a history of anti-CD20 therapy. RMD Open.

[18] Farroni C., Picchianti-Diamanti A., Aiello A., Nicastri E., Laganà B., Agrati C., Castilletti C., Meschi S., Colavita F., Cuzzi G. (2022). Kinetics of the B- and T-Cell Immune Responses After 6 Months From SARS-CoV-2 mRNA Vaccination in Patients With Rheumatoid Arthritis. Front. Immunol..

[19] Chiang T.P.-Y., Connolly C.M., A Ruddy J., Boyarsky B.J., Alejo J.L., A Werbel W., Massie A., Christopher-Stine L., Garonzik-Wang J., Segev D.L. (2021). Antibody response to the Janssen/Johnson & Johnson SARS-CoV-2 vaccine in patients with rheumatic and musculoskeletal diseases. Ann. Rheum. Dis..

[20] Prendecki M., Clarke C., Edwards H., McIntyre S., Mortimer P., Gleeson S., Martin P., Thomson T., Randell P., Shah A. (2021). Humoral and T-cell responses to SARS-CoV-2 vaccination in patients receiving immunosuppression. Ann. Rheum. Dis..

[21] Ferri C., Ursini F., Gragnani L., Raimondo V., Giuggioli D., Foti R., Caminiti M., Olivo D., Cuomo G., Visentini M. (2021). Impaired immunogenicity to COVID-19 vaccines in autoimmune systemic diseases. High prevalence of non-response in different patients’ subgroups. J. Autoimmun..

[22] Raptis C.E., Berger C.T., Ciurea A., Andrey D.O., Polysopoulos C., Lescuyer P., Maletic T., Riek M., Scherer A., von Loga I. (2022). Type of mRNA COVID-19 vaccine and immunomodulatory treatment influence humoral immunogenicity in patients with inflammatory rheumatic diseases. Front. Immunol..

[23] Picchianti-Diamanti A., Aiello A., Laganà B., Agrati C., Castilletti C., Meschi S., Farroni C., Lapa D., Fard S.N., Cuzzi G. (2021). ImmunosuppressiveTherapies Differently Modulate Humoral- and T-Cell-Specific Responses to COVID-19 mRNA Vaccine in Rheumatoid Arthritis Patients. Front. Immunol..

[24] Venerito V., Stefanizzi P., Martinelli A., Fornaro M., Galeone M.G., Tafuri S., Iannone F., Lopalco G. (2022). Anti-SARS-CoV-2 antibody decay after vaccination and immunogenicity of the booster dose of the BNT162b2 mRNA vaccine in patients with psoriatic arthritis on TNF inhibitors. Clin. Exp. Rheumatol..

[25] Frey S., Chiang T.P.-Y., Connolly C.M., Teles M., Alejo J.L., Boyarsky B.J., Christopher-Stine L., A Werbel W., Massie A.B., Segev D.L. (2022). Antibody durability 6 months after two doses of SARS-CoV-2 mRNA vaccines in patients with rheumatic and musculoskeletal disease. Lancet Rheumatol..

[26] Connolly C.M., Chiang T.P.-Y., Teles M., Frey S., Alejo J.L., Massie A., A Shah A., Albayda J., Christopher-Stine L., A Werbel W. (2022). Factors associated with poor antibody response to third-dose SARS-CoV-2 vaccination in patients with rheumatic and musculoskeletal diseases. Lancet Rheumatol..

[27] Fragoulis G.E., Karamanakos A., Arida A., Bournia V.K., Evangelatos G., Fanouriakis A., Fragiadaki K., Kravvariti E., Laskari K., Panopoulos S. (2022). Letter: Clinical outcomes of breakthrough COVID-19 after booster vaccination in patients with systemic rheumatic diseases. RMD Open.

[28] Bieber A., Sagy I., Novack L., Brikman S., Abuhasira R., Ayalon S., Novofastovski I., Abu-Shakra M., Mader R. (2022). BNT162b2 mRNA COVID-19 vaccine and booster in patients with autoimmune rheumatic diseases: A national cohort study. Ann. Rheum. Dis..

[29] Bakasis A.-D., Mavragani C.P., Voulgari P.V., Gerolymatou N., Argyropoulou O.D., Vlachoyiannopoulos P.G., Skopouli F.N., Tzioufas A.G., Moutsopoulos H.M. (2022). COVID-19: Clinical features and outcomes in unvaccinated 2-dose and 3-dose vaccinated against SARS-CoV-2 patients with systemic autoimmune and autoinflammatory rheumatic diseases. J. Autoimmun..

[30] Zhao T., Wang B., Shen J., Wei Y., Zhu Y., Tian X., Wen G., Xu B., Fu C., Xie Z. (2022). Third dose of anti-SARS-CoV-2 inactivated vaccine for patients with RA: Focusing on immunogenicity and effects of RA drugs. Front. Med. (Lausanne).

[31] Farroni C., Aiello A., Picchianti-Diamanti A., Laganà B., Petruccioli E., Agrati C., Garbuglia A.R., Meschi S., Lapa D., Cuzzi G. (2022). Booster dose of SARS-CoV-2 messenger RNA vaccines strengthens the specific immune response of patients with rheumatoid arthritis: A prospective multicenter longitudinal study. Int. J. Infect. Dis..

[32] Benucci M., Damiani A., Gobbi F.L., Lari B., Grossi V., Infantino M., Manfredi M. (2022). Role of booster with BNT162b2 mRNA in SARS-CoV-2 vaccination in patients with rheumatoid arthritis. Immunol. Res..

[33] Ferri C., Gragnani L., Raimondo V., Visentini M., Giuggioli D., Lorini S., Foti R., Cacciapaglia F., Caminiti M., Olivo D. (2022). Absent or suboptimal response to booster dose of COVID-19 vaccine in patients with autoimmune systemic diseases. J. Autoimmun..

[34] Habermann E., Gieselmann L., Tober-Lau P., Klotsche J., Albach F.N., Hagen A.T., Zernicke J., Ahmadov E., de Silva A.N.A., Frommert L.M. (2022). Pausing methotrexate prevents impairment of Omicron BA.1 and BA.2 neutralisation after COVID-19 booster vaccination. RMD Open.

[35] Stahl D., Pesch C.T., Brück C., Esser R.L., Thiele J., Di Cristanziano V., Kofler D.M. (2022). Reduced humoral response to a third dose (booster) of SARS-CoV-2 mRNA vaccines by concomitant methotrexate therapy in elderly patients with rheumatoid arthritis. RMD Open.

[36] Wroński J., Jaszczyk B., Roszkowski L., Felis-Giemza A., Bonek K., Kornatka A., Plebańczyk M., Burakowski T., Lisowska B., Kwiatkowska B. (2022). Humoral and cellular immunogenicity of COVID-19 booster dose vaccination in inflammatory arthritis patients. Front. Immunol..

[37] Mrak D., Kartnig F., Sieghart D., Simader E., Radner H., Mandl P., Göschl L., Hofer P., Deimel T., Gessl I. (2022). Accelerated waning of immune responses to a third COVID-19 vaccination in patients with immune-mediated inflammatory diseases. J. Autoimmun..

[38] Bjørlykke K.H., Ørbo H.S., Tveter A.T., Jyssum I., Sexton J., Tran T.T., E Christensen I., Kro G.B., Kvien T.K., Jahnsen J. (2023). Four SARS-CoV-2 vaccine doses or hybrid immunity in patients on immunosuppressive therapies: A Norwegian cohort study. Lancet Rheumatol..

[39] Curtis J.R., Johnson S.R., Anthony D.D., Arasaratnam R.J., Baden L.R., Bass A.R., Calabrese C., Gravallese E.M., Harpaz R., Kroger A. (2022). American College of Rheumatology Guidance for COVID-19 Vaccination in Patients With Rheumatic and Musculoskeletal Diseases: Version 5. Arthritis Rheumatol..

[40] Moss P. (2022). The T cell immune response against SARS-CoV-2. Nat. Immunol..

